# The role of PI3K-mediated AMPA receptor changes in post-conditioning of propofol in brain protection

**DOI:** 10.1186/s12868-019-0532-6

**Published:** 2019-10-01

**Authors:** Chenxu Wang, Ying Wei, Yuan Yuan, Yonghao Yu, Keliang Xie, Beibei Dong, Yuan Shi, Guolin Wang

**Affiliations:** 1 Department of Anesthesiology, Tianjin Institute of Anesthesiology, General Hospital of Tianjin Medical University, No. 154 Anshan Road, Heping District, Tianjin, 300052 People’s Republic of China; 2 Department of Anesthesiology, Tianjin People’s Hospital, Tianjin Union Medical Center, Tianjin, 300191 China

**Keywords:** Propofol, Post-conditioning, Phosphoinositide-3-kinase (PI3K), Amino-3-hydroxy- 5-methylisoxazole-4-propionic acid receptor (AMPAR), Ischemia–reperfusion injury, Rat

## Abstract

**Background:**

We aimed to study the role of amino-3-hydroxy-5-methylisoxazole-4-propionic acid receptor (AMPAR) glutamate receptor 2 (GluR2) subunit trafficking, and activity changes in short-term neuroprotection provided by propofol post-conditioning. We also aimed to determine the role of phosphoinositide-3-kinase (PI3K) in the regulation of these processes.

**Methods:**

Rats underwent 1 h of focal cerebral ischemia followed by 23 h of reperfusion were randomly divided into 6 groups (n = 36 per group): sham- operation (S), ischemia–reperfusion (IR), propofol (P group, propofol 20 mg/kg/h at the onset of reperfusion for 2 h after 60 min of occlusion), and LY294002 (PI3K non-selective antagonist) + sham (L + S, LY294002 of 1.5 mg/kg was infused 30 min before sham operation), LY294002+ ischemia–reperfusion (L + IR, LY294002 of 1.5 mg/kg was infused 30 min before middle cerebral artery occlusion), LY294002 + IR + propofol (L + P, LY294002 of 1.5 mg/kg was infused 30 min before middle cerebral artery occlusion and propofol 20 mg/kg/h at the onset of reperfusion for 2 h after 60 min of occlusion).

**Results:**

Compared with group IR, rats in group P had significant lower neurologic defect scores and infarct volume. Additionally, consistent with enhanced expression of PI3K-AMPAR GluR2 subunit complex substances in ipsilateral hippocampus, GluR2 subunits showed increased levels in both the plasma and postsynaptic membranes of neurons, while pGluR2 expression was reduced in group P. Furthermore, LY294002, the PI3K non-selective antagonist, blocked those effects.

**Conclusion:**

These observations demonstrated that propofol post-conditioning revealed acute neuroprotective role against transient MCAO in rats. The short-term neuroprotective effect was contributed by enhanced GluR2 subunits trafficking to membrane and postsynaptic membranes of neurons, as well as down-regulated the expression of pGluR2 in damaged hippocampus. Finally, the above-mentioned protective mechanism might be contributed by increased combination of PI3K to AMPAR GluR2 subunit, thus maintained the expression and activation of AMPAR GluR2 in the ipsilateral hippocampus.

## Background

With the growing population aging, there is an associated increase in the number of perioperative cerebrovascular accidents, which seriously threatens patients’ quality of life [1]. For patients presenting with a high risk of cerebrovascular diseases, choosing an appropriately safe and effective anesthetic is a highly important challenge for anesthesiologists. Taking advantage of reducing intracranial pressure and cerebral metabolic rate [2], the frequently used intravenous anesthetic, propofol, revealed its importance and potential use to such patients.

Prophase work found propofol has protective effect on rat brain from ischemia–reperfusion injury by reducing the volume of cerebral infarction, and alleviating both nerve injury and neuronal apoptosis [3]. We also studied and established the most appropriate dosage regimen for propofol (20 mg/kg/h) for post-conditioning. The mechanism behind the protection remains a major obstacle in the context of our current research. The phosphoinositide-3-kinase (PI3K)/protein kinase B (Akt) pathway plays an important role in the process of cerebral ischemia and brain protection. In the transient global cerebral ischemia model of the rat [4], the activation of the PI3K/Akt pathway may delay neuronal death in the hippocampal CA1 region [5]. When the pathway is suppressed, it can lead to the increase of DNA fragments and the increase of apoptotic neurons. In our previous study, propofol post-conditioning enhanced expression of pAkt in 24 h after transient MCAO (middle cerebral artery occlusion) [3].

Some studies showed that ischemic brain injury was closely related to amino-3-hydroxy-5- methylisoxazole- 4-propionic acid receptors (AMPAR), a tetramer system including GluR1–4 [6–8]. The continual expression of GluR2 on the cytomembrane plays a significant role in the prevention and cure of delayed neuronal injury following cerebral ischemia, due largely to its effect on decreasing permeability to Ca^2+^ by AMPARs [6, 9]. Propofol post-conditioning can maintain stable expression of GluR2 subunit in postsynaptic membranes, while the upstream regulatory mechanism has yet to be clarified. There are many molecular mechanisms and pathways that are putatively responsible for the up- or down-regulation of both subunits stability and AMPAR expression [10]. Arendt et al. [11]. concluded that the continuous low expression of PIP (3), which is the product of PI3K at the postsynaptic terminal, is necessary for stabilizing AMPARs. Additionally, Man et al. [12] determined that PI3K was key in stabilizing the migration of GluR2 in postsynaptic membranes during LTP, and did so by connecting GluR2 at the C-terminal. However, the relationship between PI3K and GluR2 during propofol induced acute post-conditioning remained ambiguous by now. Phosphorylation of GluR2 subunits at Ser880 is one of the regulatory factors of AMPA receptors membrane translocation [13].

The rats went through ischemia–reperfusion injury, and the recovery of blood-reperfusion will cause more serious injury to brain tissue. Then propofol was given after treatment, and the protective effect of propofol on brain in rats was investigated by experiment. The major focus of this study is to investigate the relationship between PI3K and GluR2 by co-immunoprecipitation, immunogold-silver staining, and Western Blotting during propofol post-conditioning. And we used LY294002, an inhibitor of PI3K, to understand the relationship between PI3K and GluR2.

## Methods

### Animals

In the experiment, we chose 216 Adult male Sprague–Dawley rats (250–280 g, provided by Tianjin Medical University), and we fed them comply with the Guide for the Care and Use of Laboratory Animals of Tianjin Medical University. All experimental operations were approved by the committee of experimental animals of Tianjin Medical University. The rats were fasting 1 day before operation, allowing free water. Rats were randomly divided into 6 groups: sham-operated group (Group S), the ischemia–reperfusion group (Group IR), and the propofol Post-conditioning group (Group P), the LY294002 group (Group L + S), the IR with LY294002 group (Group L + IR), and propofol with LY294002 group (Group L + P). LY294002 is a specific antagonist of PI3K, we used it 30 min before operation. Rats in Group S and L + S underwent a sham operation, while in Group IR and Group L + IR exposed to ischemia for 1 h and reperfusion injury for 23 h. By contrast, rats in Group P and Group L + P received propofol 20 mg/kg/h immediately after reperfusion for 2 h.

### Drugs

Propofol (Diprivan^®^) was purchased from Zeneca Company (London, UK), and was administered via femoral vein with a syringe pump (Beijing Slgo Medical Technology Development Co., Ltd., China) at a dose of 20 mg/kg/h for 2 h. For the remaining groups, equal volumes of saline were similarly administered. LY294002 was purchased from Sigma Aldrich (St. Louis, MO, USA), and dissolved in 10% dimethyl sulfoxide (DMSO, Sigma) prior to use.

### Middle cerebral artery occlusion (MCAO)

We used the intraluminal suture method for transient middle cerebral artery ischemia [14]. After the rats were anesthetized with thiobutabarbital (100 mg/kg), 1% lidocaine was given local anesthesia at the incision of the rat, we treated the nylon monofilament into the right internal carotid artery through external carotid artery. The origin of right middle cerebral artery was blocked by the monofilament, which was pushed forward 17–20 mm from the right common carotid artery bifurcation. While the monofilament was advanced less than 10 mm as sham operation. To make the reperfusion, after 1 h of ischemia, we pulled out the nylon thread for 10 mm. At the moment of reperfusion, rats received a different infusion from the right femoral vein via a TCI injection pump, while the inhibitor groups received of LY294002 at a dose of 1.5 mg/kg via intraperitoneal injection for 30 min before reperfusion. The room temperature was kept at 25–28 °C, while the rats’ rectal temperature was maintained at 37.0 ± 0.5 °C by using an electric incandescent lamp. We measured the blood pressure and arterial blood gases from polyethylene catheters inserted into the right femoral artery.

### Neurological deficits

One researcher who was unaware (blinded) of the animal grouping did the nerve injury experiments at 11 and 23 h after reperfusion. The neurological deficits were assessed on modified neurological severity score (mNSS) [15], which was composed of motor, sensation, reflex and balance ability tests. Thus, rats were evaluated for various aspects to accurately reflect the extent of nerve function damage (Table 1).

**Table 1:** Detailed description of items forming the modified neurological severity score (mNSS)

Motor tests	
Raising rat by tail (normal = 0, maximum = 3)	(3)
Flexion of forelimb	1
Flexion of hindlimb	1
Head moved > 10 degree limb vertical axis within 30 s	1
Placing rat on floor (normal = 0, maximum = 3)	(3)
Normal walk	0
Inability to walk straight	1
Circling toward the paretic side	2
Falling down to paretic side	3
Sensory tests (normal = 0, maximum = 2)	(2)
Placing test (visual and tactile test)	1
Proprioceptive test (deep sensation, pushing paw against table edge to stimulate limb muscles)	1
Beam balance tests (normal = 0, maximum = 6)	(6)
Balance with steady posture	0
Grasps side of beam	1
Huging beam and 1 limb falling down from beam	2
Huging beam and 2 limbs falling down from beam, or spins on beam (> 60 s)	3
Attempting to balance on beam but falling of (> 40 s)	4
Attempting to balance on beam but falling of (> 20 s)	5
Falling of; no attempt to balance or hang on the beam (< 20 s)	6
Reflex absence and abnormal movements (normal = 0, maximum = 4)	(4)
Pinna reflex (head shaken when auditory meatus is touched)	1
Corneal reflex (eyes blink when cornea is lightly touched with cotton)	1
Startle reflex (motor response to brief noise from clapping hands)	1
Seizures, myoclonus, myodystony	1
Maximum points	(18)

One point is given for an absent reflex tested or for the animal’s inability to perform a task: 1–6 mild injury, 7–12 moderate injury, and 13–18 severe injury

### Measurement of cerebral infarction volume

At 11 h and 23 h after reperfusion, we anesthetized the rats (pentobarbital at 130 mg/kg for intraperitoneal injection) and took out brains (6 rats at each time node in each group). The steps are as follows: first cut off the head with large scissors, quickly trimmed off the muscles around the occipital bone, then pried open the occipital bone with vascular forceps, and carefully pried open the parietal bone on both sides of the brain, detached it from both sides of the brain with curved tweezers, and lifted the brain from the front. The brains were then cut into 6 coronal slices of 2 mm. The slices were soaked in 2% triphenyltetrazolium chloride (TTC) light sheltered for 30 min at 37 °C, and then fixed in 4% paraformaldehyde overnight. We took pictures of the back of each slice and analyzed the infarct size with AutoCAD. The cerebral ischemic volume was calculated by multiplying the area by the thickness (2 mm). To reduce the effects of edema, we adopted the following formula as previously described [16, 17]: Corrected Infarct Volume = Contralateral Hemisphere Volume − (Ipsilateral Hemisphere Volume-Measured Infarct Volume).

### Tissue preparation

The expression of GluR2 subunits and pGluR2 were investigated by Western blotting. We have previously confirmed the specificity of antibodies to GluR2, whereas the specificity to pGluR2 was tested in this study by Western blotting assay. Hippocampus of the ischemic hemisphere and containing GluR2 subunits were obtained 5 h, 11 h, and 23 h after MCAO. After being anesthetized by pentobarbital at 130 mg/kg for intraperitoneal injection, the rat’s brain was removed and the right hemisphere was separated longitudinally. Then we cut off brain tissue, fed primarily by the anterior cerebral artery, approximately 2 mm from the midline. The injured hippocampus was exposed and stripped, and cut. Tissues (n = 4–5, in each group) were shredded and then proteins were isolated by lysis buffer (50 mmol/L Tris–HCl pH = 6.8, 0.5% sodium deoxycholate, 0.1% SDS, 150 mmol/L NaCl, 0.5% NP-40, 5 mmol/L EDTA, protease inhibitor cocktail), followed by centrifugation (2000*g*, 5 min, at 4 °C). When it comes to the GluR2 on the membrane, we used a eukaryotic membrane protein extraction reagent kit (Mem-PER™ Eukaryotic Membrane Protein Extraction ReagentKit, Pierce Biotechnology, Rockford, IL, USA)to extract GluR2 located on membrane from the total GluRs. The right hippocampus was sliced up and made tissue grinder with TBS. The homogenate was centrifuged and as the instructions of kits indicated incubated with reagent A, B, and C sequentially (Mem-PER™ Eukaryotic Membrane Protein Extraction ReagentKit, Pierce Biotechnology, Rockford, IL, USA). After centrifuge, tissues would be isolated the hydrophobic fraction, among which the hydrophilic phase was what we need and the majority of membrane protein would be in the lower hydrophobic fraction. A commercial BCA Protein Assay reagent kit (Pierce, Rockford, IL, USA) was used to measure total and membrane protein concentrations.

### Western blot and co-immunoprecipitation

The protein used for co-immunoprecipitation was freshly prepared while that used for Western blot was stored at − 80 °C until assayed. The PI3K–GluR2 complex was detected by co-immunoprecipitation. As Yang et al. described [18], protein solution was centrifuged, and the supernatants were selected by protein A/G beads (Santa Cruz Biotechnology, Santa Cruz, CA, USA) and immunoprecipitated overnight at 4 °C with anti-GluR2 (rabbit monoclonal; Millipore) antibodies. The complex substance was precipitated in on protein A/G beads and washed in IP buffer for 5 times (IP buffer: 50 mM Tris–HCl, pH = 7.4, 0.5% Nonidet P-40, 300 mM NaCl), followed by cold PBS at 4 °C once. Both the precipitate and protein lysates were boiled in Laemmli buffer at 95 °C for 5 min (Laemmli buffer: 60 mM Tris–HCl, pH = 6.8, 10% glycerol, 2% SDS, 5% β-mercaptoethanol, and 0.01% bromphenol blue) for Western blot analysis. We used the procedures outlined by Adotevi et al. [19] whereupon the proteins were separated by SDS-PAGE and then transferred onto PVDF membranes (Millipore). Specified antibodies were dissolved in TBST buffer (100 mM Tris–HCl, pH = 7.5, 150 mM NaCl, 0.1% Tween 20) with 5% skimmed milk powder (Tesco) and ECL (GE Healthcare) for detecting. The antibodies included anti-GluR2 (1:1000, monoclonal, CST, USA) antibody for measuring the expression of total GluR2 (110 kDa), anti-pGluR2 (1:1000, monoclonal, Abcam, USA) antibody for detecting the expression of pGluR2, and anti-PI3K (1:1500, monoclonal, CST, USA) antibody for investigating the expression of the PI3K–GluR2 complex (85 kDa) in the immunoprecipitation assay. Blots were washed and then incubated with goat anti-rabbit IgG secondary antibody conjugated to horseradish peroxidase (1:5000, Santa Cruz Biotechnology, CA, USA) for 1 h at room temperature and signals were displayed by ECL (Amersham, Buckinghamshire, UK). Quantitative analysis of the band densities was performed by Quantity One Analyzer 4.5 (BioRad Laboratories, CA) in a blinded fashion, semi-quantitative determination of the levels of both GluR2 and pGluR2 were reflected by the percentages of light densities as compared with β-actin, while the complex of PI3K–GluR2 were compared with that of GluR2.

### Immunogold-silver staining and electron microscope

Following 23 h of reperfusion, the animals in Group S, IR, P and L + P (n = 6 in each group) were anesthetized with pentobarbital at 130 mg/kg and injected with 1000 IU heparin into the aorta. Thereafter, and according to the procedures outlined by Sonomura et al. [20], rats were perfused with 200 ml of saline solution followed by 100 ml of fixative solution supplemented with 2% paraformaldehyde (Sigma, USA) and 3.8% acryl aldehyde (Sigma, USA) in 0.1 M PBS, pH = 7.4 through the left ventricle and at room temperature. Next, the brain was removed and immersed overnight in 2% paraformaldehyde fixative solution. The right hemisphere of the brain enabled coronal sections (30–40 μm) to be obtained using a vibratome (Leica, Germany), which were then immersed in 1% sodium borohydride in 0.1 M PBS for 30 min. Tissue sections were then rinsed in 0.1 M PBS, followed by 0.1 M Tris-buffered saline (TBS; pH = 7.6) and blocked in 1% bovine serum albumin (BSA) in TBS before incubation in respective primary antibodies.

Tissue slices were incubated overnight at 4 °C in GluR2 subunit primary antibody (1:500, monoclonal, Abcam, USA) in 0.1 M TBS with 0.1% BSA. For immunogold-silver staining of GluR2 trafficking, slices were incubated in 1 nM gold-conjugated goat anti-rabbit IgG (1:50, Amersham Corp., Amersham, England), and enhanced staining with a silver enhancement kit (Amersham Corp.), then fixed in 2% glutaraldehyde in PBS. The ischemic was collected on copper mesh grids and then sliced into ultrathin sections hippocampus, and photographed by a Morgagni 268 (FEI Company, Hillsboro, Oregon) digital electron microscope. Adobe Photoshop (Adobe, San Jose, CA) was used to regulate brightness and contrast and to form the final images. 9 neurons and 5 cynapsis around were chosen randomly and the percentages of GluR2 subunits in the postsynaptic membrane were calculated by counting the positive granules in both cytoplasm and plasma membrane to reflect the trafficking of GluR2 subunits.

### Statistical analyses

Data are presented as mean ± SEM and analyzed by SPSS version 18.0 statistical software. Infarct volume, co-immunoprecipitation, and Western blot data were analyzed by multivariate analysis of variance (MANOVA), while mNSS scores were analyzed by repeated measures analysis of variance (ANOVA). Statistical analyses of immunogold-silver staining were performed using one-way ANOVA. The individual comparisons of means were analyzed by post hoc LSD tests. All values were considered statistically significant for alpha values of P < 0.05.

## Results

### Physiological variables of rats

We monitored physiological parameters that consisted of mean arterial blood pressure, glucose, and blood gas analysis during the experiment (done at three time-points: 10 min before occlusion, 1 h after occlusion, and 1 h after reperfusion). No statistical differences were observed in these physiological parameters and blood gas analysis among the rats in different groups (the results of blood gas were showed in Table 2). There were no adverse events such as anoxia and asphyxia caused by operation and propofol infusion.

**Table 2:** The results of blood gas in different group

Group	10 min before occlusion				1 h after occlusion				1 h after reperfusion			
	PH	PO_2_	PCO_2_	SO_2_	PH	PO_2_	PCO_2_	SO_2_	PH	PO_2_	PCO_2_	SO_2_
S	7.38 ± 0.057	81.44 ± 5.008	26.07 ± 2.099	98.08 ± 1.627	7.34 ± 0.060	82.74 ± 4.981	25.13 ± 2.382	96.93 ± 2.827	7.33 ± 0.065	80.78 ± 5.205	25.86 ± 2.938	95.34 ± 3.340
L ± S	7.42 ± 0.027	82.03 ± 3.840	25.16 ± 2.788	96.93 ± 1.900	7.32 ± 0.096	81.85 ± 5.515	24.26 ± 3.041	96.41 ± 3.137	7.31 ± 0.075	83.29 ± 3.335	26.16 ± 2.272	97.35 ± 2.420
IR	7.40 ± 0.062	82.98 ± 4.784	25.33 ± 1.802	97.63 ± 2.396	7.33 ± 0.065	83.76 ± 4.820	25.80 ± 2.529	97.58 ± 2.160	7.32 ± 0.068	82.16 ± 4.058	24.94 ± 2.144	97.05 ± 2.208
L ± IR	7.38 ± 0.064	81.15 ± 5.521	25.43 ± 2.421	96.48 ± 2.458	7.39 ± 0.033	82.22 ± 6.423	25.50 ± 2.231	96.43 ± 2.570	7.34 ± 0.039	83.12 ± 3.859	25.79 ± 2.645	97.30 ± 2.196
P	7.42 ± 0.054	80.33 ± 7.733	27.21 ± 2.421	97.55 ± 2.377	7.40 ± 0.071	81.75 ± 5.425	24.42 ± 1.890	97.23 ± 1.392	7.39 ± 0.059	80.25 ± 4.857	26.06 ± 2.949	96.08 ± 1.365
L+P	7.41 ± 0.060	82.30 ± 4.061	24.72 ± 2.831	96.88 ± 2.238	7.31 ± 0.062	82.06 ± 6.233	24.25 ± 1.748	97.27 ± 1.512	7.34 ± 0.035	81.96 ± 4.244	26.29 ± 1.552	96.08 ± 2.184

### Propofol post-conditioning of neurological deficit after IR injury in rats

**Fig. 1:**
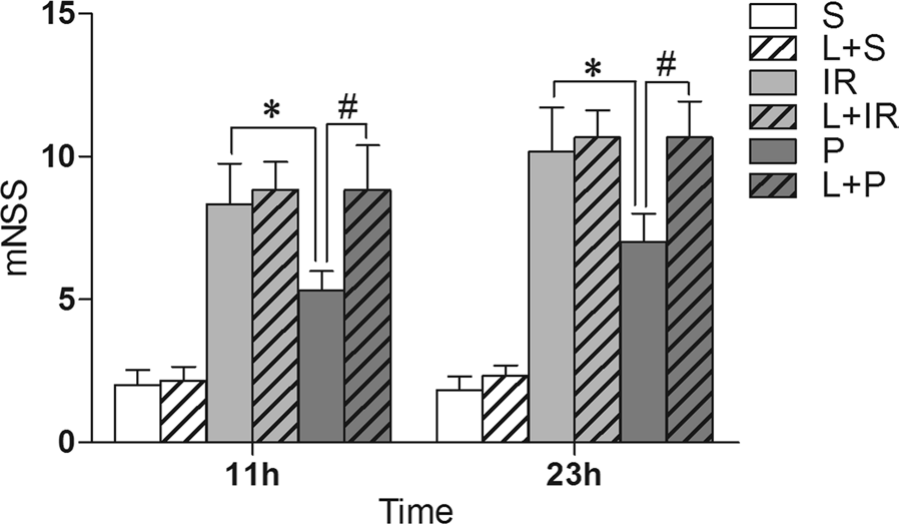
Propofol post-conditioning reduced the behavior deficit. Behavior tests showed propofol post-conditioning significantly improved function outcome. The mNSS in groups received propofol post-conditioning is significantly less than that in IR Group both at 11 h (P = 0.035) and 23 h (P = 0.042) after IR injury. Administration of LY294002 partly but significantly reversed the decrease in scores induced by propofol (P = 0.015 at 11 h, and P = 0.020 at 23 h after IR injury). n = 6 for each group. S: sham-operated group; IR: P: propofol Post-cond group; L + S: sham-operated group; L + IR: LY294002 + ischemia–reperfusion injury group; L + P: LY294002 + propofol Post-cond group. *P < 0.05 vs Group IR; ^#^P < 0.05 vs Group P

In the following 23 h of reperfusion after ischemic injury, we measured the modified neurological severity score (mNSS). A lower score showed rats had lower neurological defects from IR injury (P < 0.0001 at both 11 h and 23 h compared with Group S), and presented with more improved outcome by propofol post-conditioning. The statistical data showed that propofol at the dose of 20 mg/kg/h infusion for 2 h significantly decreased the neurological deficit as compared with group IR at both 11 h (P = 0.035) and 23 h (P = 0.042) after IR injury. Administration of LY294002 partly but significantly reversed the decrease in scores induced by propofol (P = 0.015 at 11 h, and P = 0.020 at 23 h after IR injury, Fig. 1).

### Propofol treatment on infarct volume in rats with IR injury

**Fig. 2:**
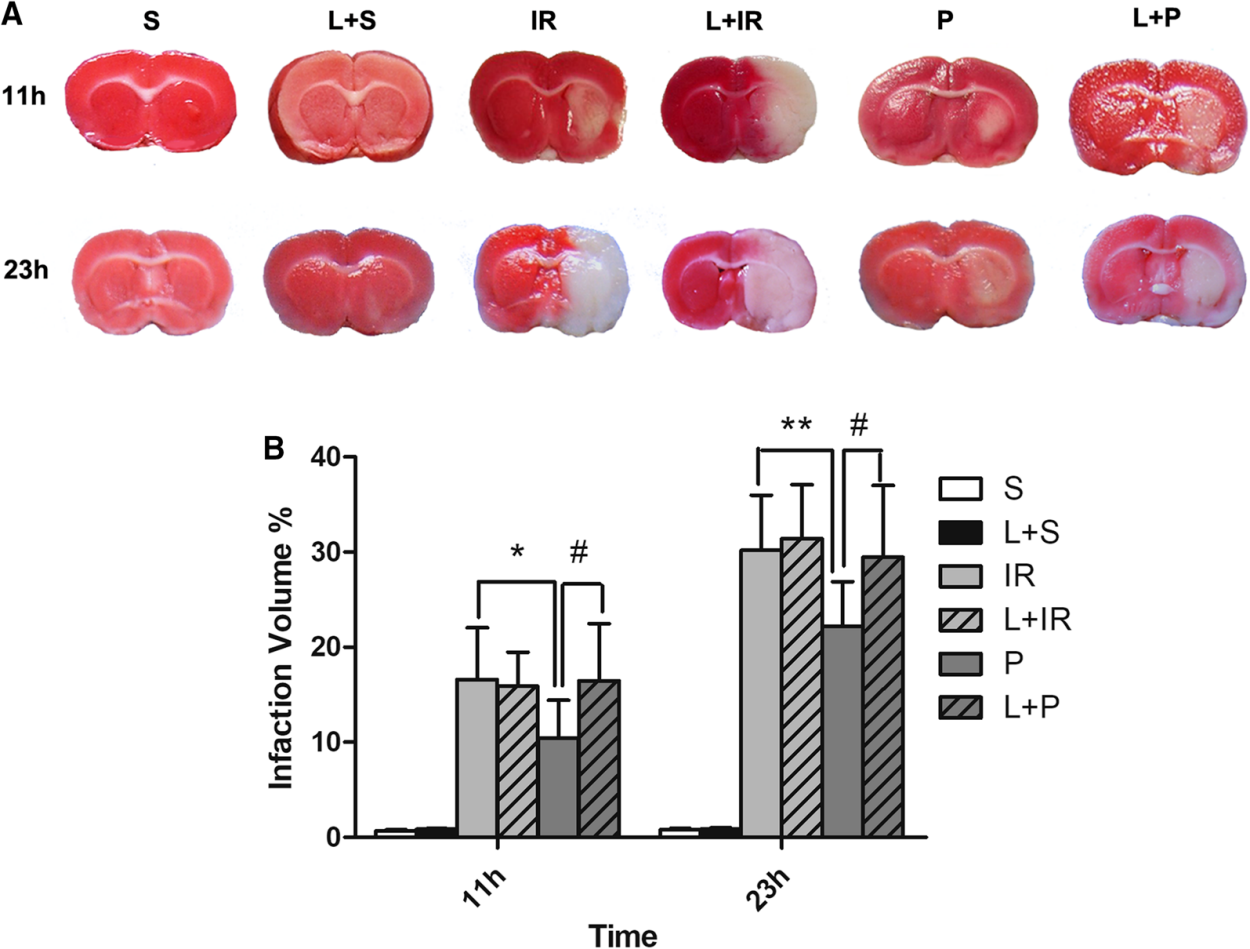
Propofol post-conditioning mitigated the infarction injury. **A** Pictures of TTC staining in brain sections. **B** Statistical results of cerebral infarction volume in each group of rats. Infarcted area shown on TTC staining (white color) was prominent in the IR Group while decreased in propofol treated groups at two time points (P = 0.12 at 11 h and P = 0.008 at 23 h after IR injury). By contrast, pretreatment with LY294002 partly eliminated the protection induced by propofol (P = 0.014 at 11 h and P = 0.016 at 23 h as compared with group P, respectively). n = 6 for each group. S: sham-operated group; IR: P: propofol Post-cond group; L + S: sham-operated group; L + IR: LY294002 + ischemia–reperfusion injury group; L + P: LY294002 + propofol Post-cond group.*P < 0.05 vs Group IR; **P < 0.01 vs Group IR; ^#^P < 0.05 vs Group P

The percentage of infarct size in brain was calculated by TTC staining of brain slices. All the groups presented infarct lesion, which differentially became gradually larger with the exception of Group S and L + S at 23 h after IR injury. We found that the infarct volume at both time-points decreased significantly in group P as compared with group IR (P = 0.12 at 11 h and P = 0.008 at 23 h after IR injury). By contrast, pretreatment with LY294002 partly eliminated the protection induced by propofol (P = 0.014 at 11 h and P = 0.016 at 23 h as compared with group P, respectively, Fig. 2a, b).

### Propofol post-conditioning effect on the expression of the PI3K and GluR2 complex

**Fig. 3:**
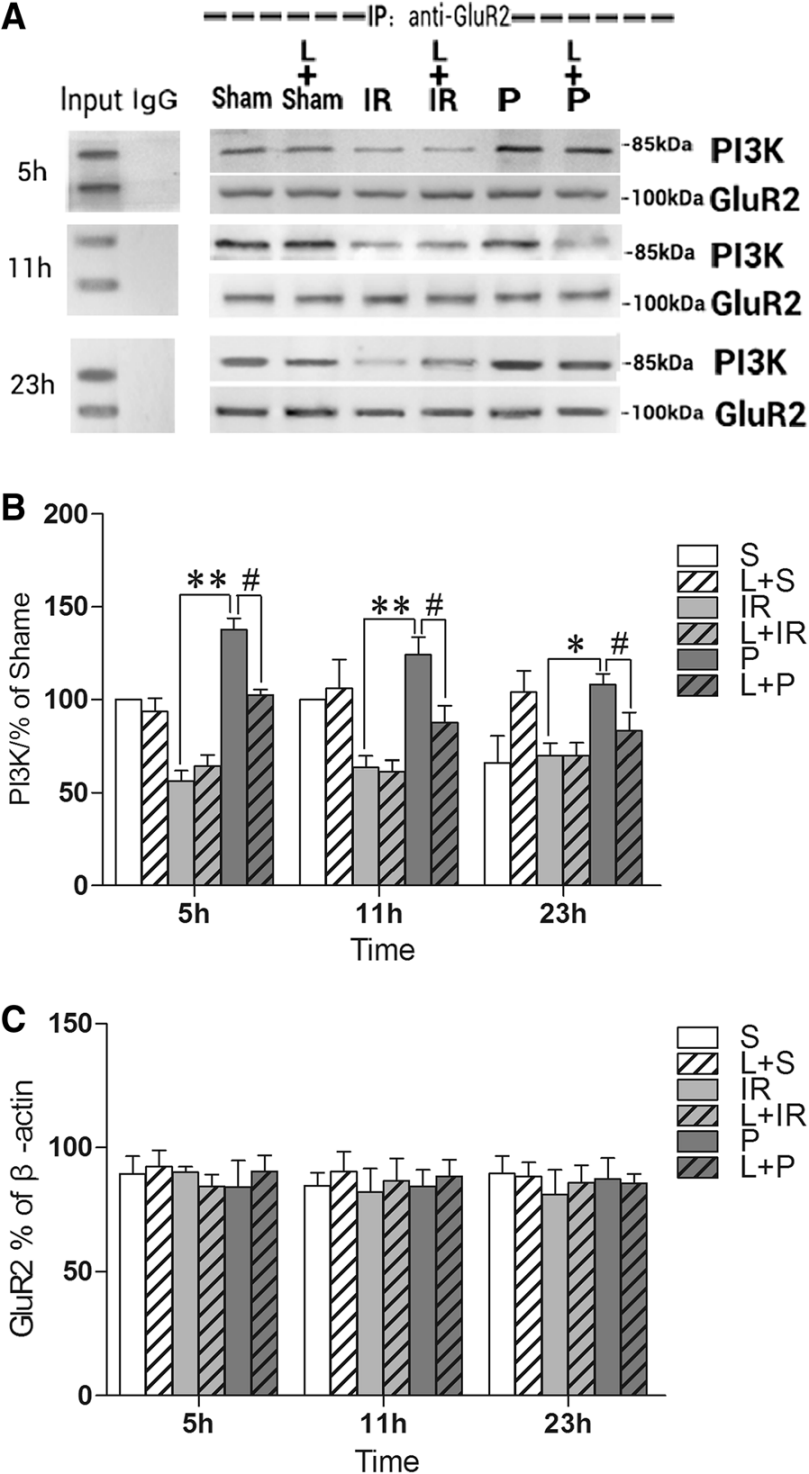
Propofol post-conditioning enhanced the combination of GluR2 and PI3K. **A** The western blotting assay of PI3K–GluR2 complex. The tissue from ischemic hippocampus was incubated with antibody of GluR2. The complexes were collected on protein A/G beads. Anti-PI3K antibody was used for investigating the expression of PI3K–GluR2 complex in the immunoprecipitation at 85 kDa. Anti-GluR2 antibody was to detect the concentration of total GluR2 which reflected at 100 kDa. S: sham-operated group; IR: P: propofol Post-cond group; L + S: sham-operated group; L + IR: LY294002 + ischemia–reperfusion injury group; L + P: LY294002 + propofol Post-cond group. **B** The statistical result of optical density was gathered and compared with Shame Group to get the results of complex. compared S Group, other groups at any time point had more expression of PI3K–GluR2 complex; while in Group P the complex containing was higher compared with Group IR (P < 0.0001, P < 0.0001, and P = 0.001 at each time-point).; applied LY294002 depressed the trend which made by propofol (P = 0.015, P = 0.011, and P = 0.023 at each time-point). n = 6 for each group.*P < 0.05 vs Group IR; **P < 0.01 vs Group IR; ^#^P < 0.05 vs Group P. **C** The expression of total GluR2. There were no significant differences in each group at each time point

Co-immunoprecipitation analysis was to reveal the effect of propofol post-conditioning on co-expression of PI3K and GluR2 at 5 h, 11 h and 23 h after IR injury. Total GluR2 expression in the ischemic hippocampus was comparable in all groups after 23 h of reperfusion (P > 0.05). Infusion of propofol for 2 h significantly increased the PI3K and GluR2 complex level in the ischemic hemicerebrum of rats as compared with the IR group (P < 0.0001, P < 0.0001, and P = 0.001 at each time-point). Administration of the PI3K inhibitor, LY294002 partly reversed the elevated levels of the complex (P = 0.015, P = 0.011, and P = 0.023 at each time-point) as compared group P (Fig. 3a, b).

### Effect of propofol on trafficking of GluR2 subunits in IR injured rats

**Fig. 4:**
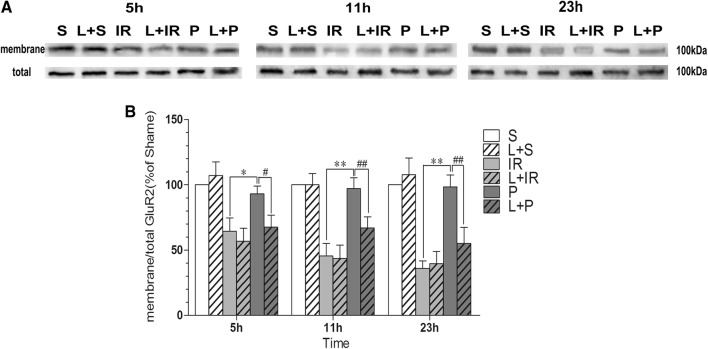
Propofol post-conditioning promoted the expression of GluR2 on the membrane structure. **A** The tissue extracted by the membrane protein kit was reflected by Western Blotting with antibody of GluR2 subunit. In Group IR, most of the GluR2 located on the membrane structure, while in L + P Group the trafficking to membrane structure was weakened. S: sham-operated group; IR: P: propofol Post-cond group; L + S: sham-operated group; L + IR: LY294002 + ischemia–reperfusion injury group; L + P: LY294002 + propofol Post-cond group. **B** We measured the concentration of GluR2 subunits located on membrane with the proportion of M/T (membrane/total) and found that the proportion in Sham Group did not change much, so the results in other groups were reflected by the comparison with Sham group. Compared with Group IR the GluR2 moved to the membrane structure (P = 0.015 at 5 h, P < 0.0001 at 11 h and 23 h). Compared with Group P, the ratio of M/T in Group L + P was lower (P = 0.028 at 5 h, P = 0.005 at 11 h, P = 0.002 at 23 h). n = 6 for each group.*P < 0.05 vs Group IR; **P < 0.01 vs Group IR; ^#^P < 0.05 vs Group P; ^##^P < 0.05 vs Group P

We used antibody of GluR2 to detect the expression of membrane structure (M) and total (T) AMPA receptor GluR2 reflected by Western Blotting (Fig. 4a, b). The expression of total GluR2 were almost same in every group (P > 0.05) while the membrane values had different levels. The distribution of GluR2 was described by the ratio of M/T compared with S Group at each time point (Fig. 4a, b). Rats in the IR Group had significantly low GluR2 M/T values compared with sham-operated rats (P < 0.0001 at every tome point), which means the IR injury caused the GluR2 subunits internalization to the cytoplasm from the membrane structure. The situation were changed when the rats were under the post-conditioning of propofol, for which in Group P the ratio was significantly higher than that in IR Group (P = 0.015 at 5 h, P < 0.0001 at 11 h and 23 h). The changes of the distribution showed that propofol post-conditioning strengthened the GluR2 subunits located on the membrane structure in hippocampus. When the inhibitor of PI3K was administered, some of the membrane GluR2 subunits moved to the cytoplasm and made the M/T ratio lower compared with P Group (P = 0.028 at 5 h, P = 0.005 at 11 h, P = 0.002 at 23 h). LY294002 might influence the distribution of GluR2, which means the PI3K might be the upstream of AMPA receptor GluR2 subunits. While the membrane structure in nerve cells was composed of cytomembrane, membranous organelles and cytoskeleton, only Western Blotting results can’t explain the location of GluR2 at subcellular level. Then we observed the exact location from electron microscope pictures.

**Fig. 5:**
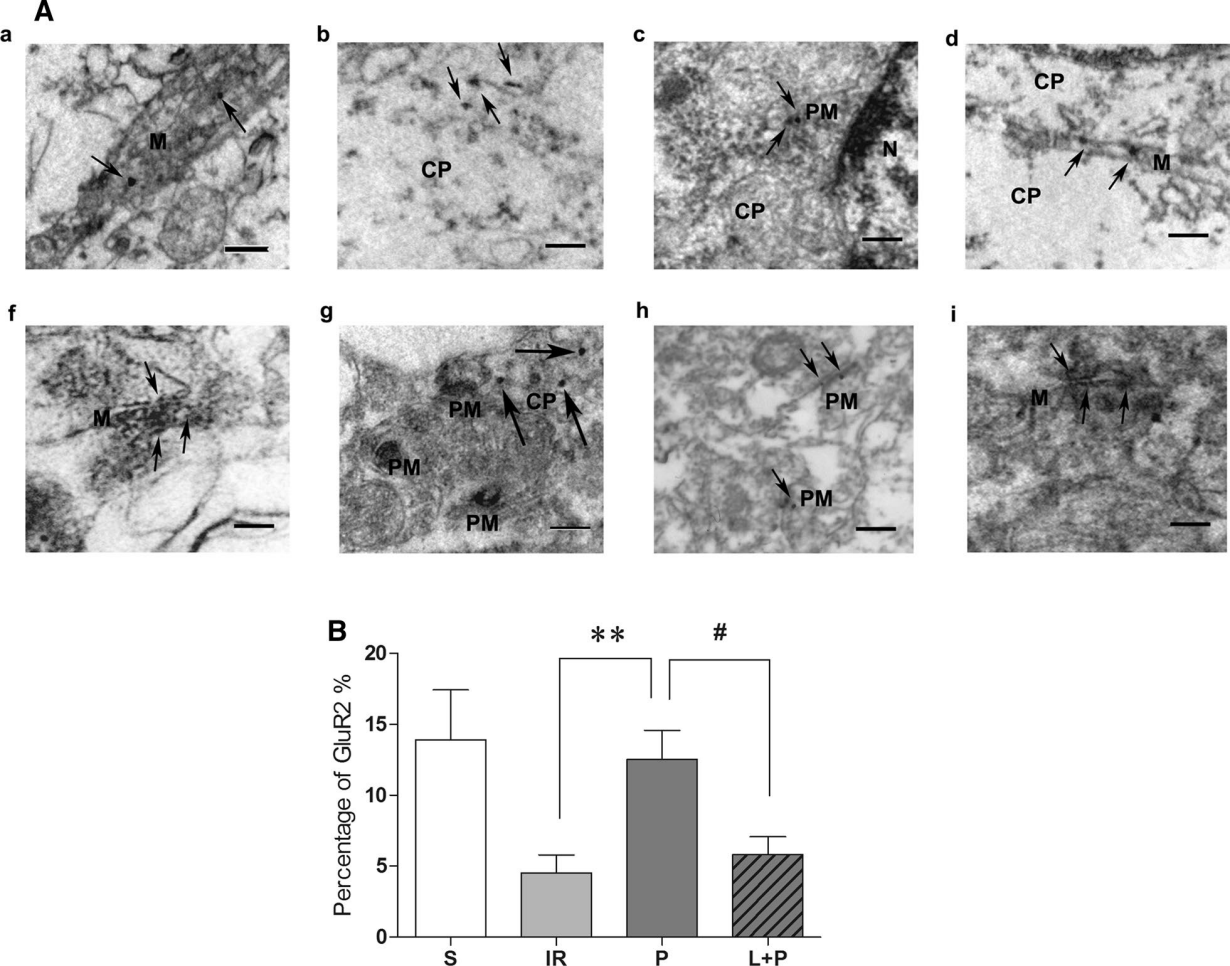
Propofol post-conditioning promoted GluR2 trafficking to the postsynaptic membrane of neurons. **A** Electron micrographs of ischemic hippocampus from rats with IR injury (**b**, **g**), propofol post-conditioning (**c**, **h**), and Sham operation (**a**, **f**), LY294002 treated (**d**, **i**). In the S Group, some of GluR2 subunits located on cytomembrane (**a**) while some of them located on other membrane structure, such as mitochondria or endoplasmic reticulum (**f**). When the brain was suffered from IR injury, the GluR2 moved to the plasma (**b**, **g**); while propofol was given, most of GluR2 subunits returned back to the cytomembrane and postsynaptic membrane (**c**, **h**). In the LY294002 treated tissue, the GluR2 subunits is rare on the postsynaptic membrane but other membrane structure (**d**, **i**). The arrows showed the positive granules of GluR2 whose diameter was 1.4 nm and with lower transparency. M, cytomembrane; CP, cytoplasm; PM, postsynaptic membrane. *Scale bars:* **a**, **b**, **c**, **d**, **g** = 4 nm; f, h, i = 40 nm. **B** 9 neurons and 5 synapsis around were chosen randomly and the percentages of GluR2 subunits in the postsynaptic membrane were calculated by counting the positive granules in both cytoplasm and plasma membrane to reflect the trafficking of GluR2 subunits. In Group P, GluR2 located on postsynaptic membrane was more than that in Group IR (P = 0.20). S: sham-operated group; IR: P: propofol Post-cond group; L + P: LY294002 + propofol Post-cond group. n = 6 for each group. **P < 0.01 vs Group IR; ^#^P < 0.05 vs Group P

Electron microscopic photographs are used to describe subcellular localization of GluR2 subunits in the ischemic hippocampus of rats that underwent 1 h of ischemia and 23 h of reperfusion injury. Black deposits indicated gold-silver immunolabeling were easy to distinguished from the electron dense peroxidase reaction products. The diameters of the granules were almost 1.4 nm (as shown by the arrows in Fig. 5a). In each section, 9 neurons and 5 synapses were chosen randomly, and 5 sections were used to calculate the positive granules in both the cytoplasm and plasma membrane to reflect trafficking of GluR2 subunits in each hippocampus.

Semi-quantitative analysis was used to calculate the percentage of GluR2 subunits localized to the membrane of neurons in the cerebral hemispheres of rats subjected to IR injury and treated with or without propofol post-conditioning or the inhibitor LY294002. In the rat of by sham operation, the GluR2 subunits were localized on both cytoplasmic membrane and intracellular compartments of neurons, and the majority located on plasma membrane (almost 14%). When subjected to IR injury, the majority of GluR2 subunits were found localized in the plasmalemma and intracellular compartments, such as the mitochondria (Fig. 5a), and down-regulated on the cytoplasmic membrane or synapse/postsynaptic membrane. Additionally, the percentage of GluR2 subunits located in the postsynaptic membrane was 4.5% (P = 0.007 as compared with Group S). Propofol at a dose of 20 mg/kg/h post-conditioning redistributed GluR2 to the postsynaptic membrane. This was especially evidents compared with Group IR (12.5% vs. 4.5%, P = 0.02, Fig. 5b). Moreover, in Group L + P, reduced frequencies of the GluR2 subunits were found in the postsynaptic membranes (5.81%) as compared with that in Group P (P = 0.046).

### The expression of pGluR2 during post-conditioning induced by propofol

**Fig. 6:**
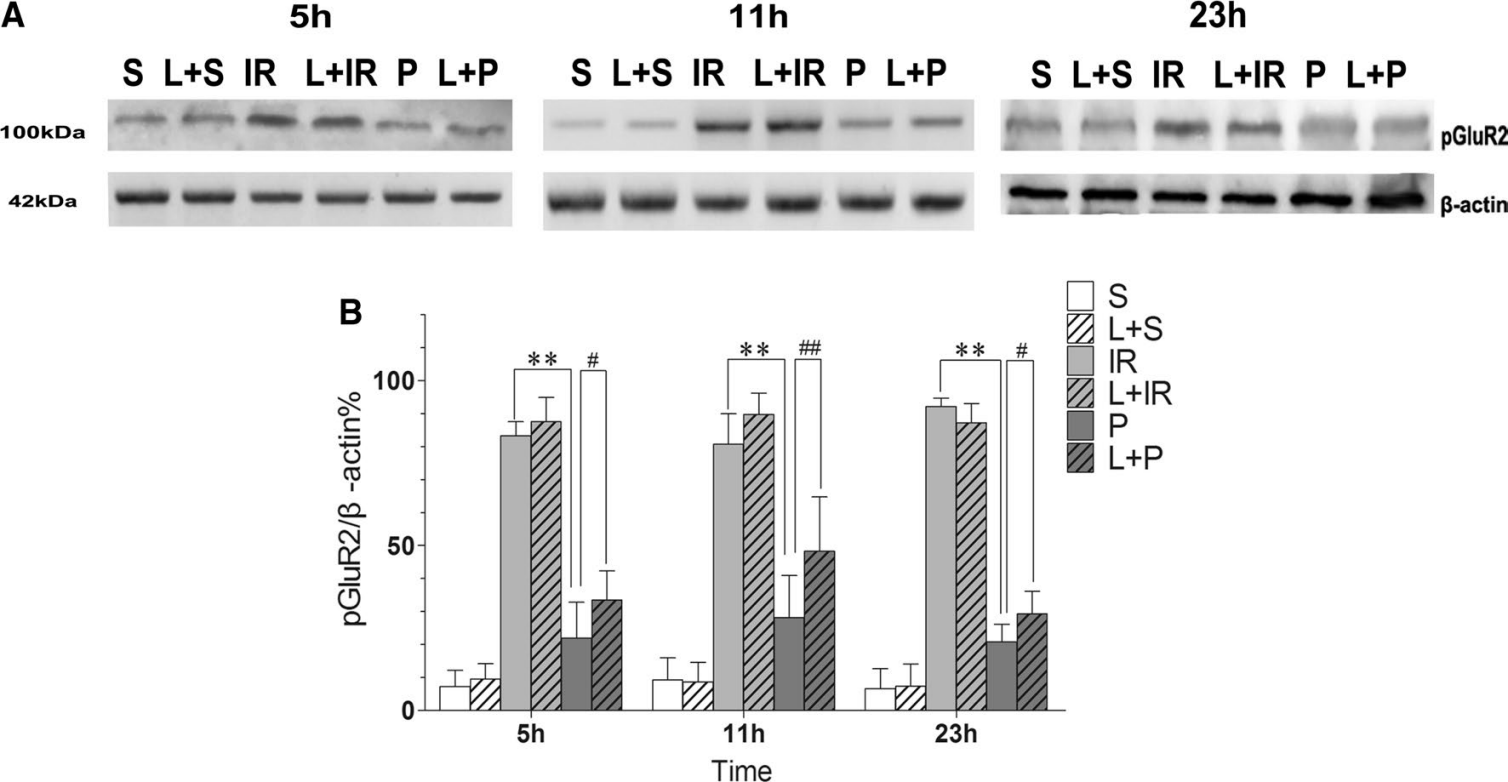
The expression of pGluR2 during post-conditioning induced by propofol. **A** The phosphorylation level of GluR2 after propofol post-conditioning. The tissue from Ischemic hippocampus was incubated with anti-pGluR2 antibody (S880) and concentration of pGluR2 was detected by Western Blot (100 kDa). S: sham-operated group; IR: P: propofol Post-cond group; L + S: sham-operated group; L + IR: LY294002 + ischemia–reperfusion injury group; L + P:LY294002 + propofol Post-cond group. **B** Compared with Group S, the injury brain would express more pGluR2;when compared with Group IR, it was at lower level in Group P; More noteworthy is that inhibitor of PI3K could up-regulate the level which was reduced by propofol. n = 6 for each group. **P < 0.01 vs Group IR; ^#^P < 0.05 vs Group P; ^##^P < 0.05 vs Group P

We measured the levels of pGluR2 in the ischemic hippocampus by Western blot assay. The results were reflected by the ratio of light densities relative to that of total GluR2. As compared with Group S, the rats in the other groups expressed more pGluR2 (P < 0.01), which meant that IR injury might have provoked the phosphorylation of GluR2. In Group P, the extent of phosphorylation was attenuated by propofol post-conditioning (20 mg/kg/h) as compared with that in Group IR over the following 23 h (P = 0.0001 at each time-point). Additionally, LY294002 played an important role in promoting significant accumulation of pGluR2 as compared that seen in Group P (P = 0.010, P = 0.002, and P = 0.016 at each time-point). However, the expression of pGluR2 at different time-points was no different between Groups IR and L + IR (P > 0.05, Fig. 6a, b).

## Discussion

This research clarified that propofol (20 mg/kg/h) post-conditioning for 2 h at the onset of reperfusion (ischemia for 1 h) can provide acute (23 h) neuroprotection against IR injury induced by MCAO in rats. Both histological and neurological data can support experimental conclusions, including a reduction in the brain infarct volume and attenuation of the neurological deficit. We also showed that propofol (20 mg/kg/h) for 2 h post-conditioning differentially regulated the co-expression of PI3K and GluR2 subunits, since a marked increase in the PI3K–GluR2 complex was observed in the ischemic hippocampus, which contributed to the trafficking of GluR2 subunits in postsynaptic membrane (as observed by Western blotting assay and electron microscopy staining). The down-regulated level of phosphorylated GluR2 subunits was also one of the results induced by the co-expression of PI3K and GluR2 and contributed to propofol post-conditioning.

### The GluR2 subunits and AMPA receptors

AMPA receptors (AMPAR) are involved in a lot of physiological processes in mature or developing brain, including synaptic plasticity, learning and memory formation [21, 22]. AMPARs mediate almost 70% of the excitatory synapse transmissions in CNS [23]. Additionally, the nerve damage and pathogenesis of neuronal loss caused by cerebral ischemia is also mediated by AMPAR [24, 25]. Some experiments have indicated that AMPAR can lead to the increase of intracellular Ca^2+^and Zn^2+^ concentration, resulting in cytotoxic changes and nerve injury [26–28], with subsequent activation of diverse downstream cell death signals. Although the underlining mechanisms are not fully clear, it is suggested that Ca^2+^-permeability of AMPARs varies depending on whether the GluR2 subunits are present within the AMPARs [29]. AMPARs are tetrameric assemblies of different combinations of four homologous subunits designated GluR1-GluR4. The ion selectivity of AMPARs containing GluR2 subunits is dominant over other subunits. It is now clear that there exist AMPARs without GluR2 subunits exhibiting considerable Ca^2+^ permeability, but when they contain GluR2 subunits the permeability to Ca^2+^ becomes very scarce [27, 29, 30]. The internalization of GluR2 subunits may lead to the influx of Ca^2+^, which may lead to nerve damage in the ischemic area [30, 31]. And the changes in the distribution of GluR2 subunits can cause activation of a caspase-dependent apoptotic pathway [24]. Thus, stabilization of GluR2 plays an important role in prevention and cure of neuronal injury. However, the changes to the trafficking and activity of GluR2 subunits are still indeterminate, as are the mechanisms responsible for upstream regulation.

### The trafficking of GluR2 subunit-containing AMPAR

The movement of AMPAR between cell membrane and cytoplasm is a dynamic process and is mediated by a variety of proteins [32] that always bind to GluR2 and GluR3 subunits, thereby mediating the long-term potentiation (LTP) and long-term depression (LTD) [33]. Studies by Man et al. [12] further revealed that GluR2-associated PI3K activity mediated AMPAR trafficking. This model showed that, Ca^2+^ influx via activated NMDAR. High concentration of Ca^2+^ results in activation of a GluR2-associated PI3K and increased expression of membrane bound phosphoinositide-3,4,5-trisphosphate (PtdIns-3P), which will facilitate membrane targeting of AMPARs. Investigating the changes of GluR2 was one of our research objectives, as the Western Blotting results showed that the total GluR2 subunits did not change much after IR injury and propofol post-conditioning, while on the membrane structures the expression of GluR2 was up-regulated, the trafficking of GluR2 deserved investigation. Eukaryotic membrane protein extraction kit selected the protein on membrane, but GluR2 might located on diverse intracellular organelles, such as lysosomes, mitochondria, and Golgi Complexes. A great deal of the subcellular structures are membranous despite the protein on membrane was extracted by cross-linking assay, the precise location of GluR2 still need intuitive images to reveal. So immunogold-silver staining was for the further study of the GluR2 trafficking on the postsynaptic membrane. At 23 h after reperfusion, the expression of GluR2 subunit changed obviously on the membrane, so we did only one time point of electron microscopic observation, and we chose only four groups of animals for comparison. The rest of the time points and animal groups need to be discussed in further experiments. As we showed in our current study, propofol post-conditioning might stabilize the GluR2 subunits expression on postsynaptic membrane rather than other membrane structure, which would depress the Ca^2+^-permeability of AMPARs with GluR2 subunits.

### The phosphorylation of GluR2 subunits

AMPAR GluR2 subunits contain postsynaptic density protein 95/discsslarge/zon occlusens-1 (PDZ) domain, which can be phosphorylated at serine 880 (S880) [34] and is responsible for the trafficking and activity of AMPARs. It does so by disrupting interactions of GluR2 subunits with its synaptic anchoring proteins, removing synaptic GluR2 subunits, and mediating some form of activity-dependent synaptic depression [35]. In addition, phosphorylation of S880 in synaptic GluR2 subunits can induce the redistribution of AMPARs away from the synapses, which will cause receptor endocytosis, as during long-term depression (LTD). In this study, the majority of AMPARs with phosphorylating GluR2 subunits remained settling on the postsynaptic membrane and did not co-traffick with the endocytosed receptor population. By contrast, the non-phosphorylated receptors co-trafficked with the endocytosed receptors. Phosphorylation of S880 on GluR2 subunits is also closely related to brain degenerative diseases as well as the trafficking of GluR2 subunits. Studies that have focused on the pathogenesis of Alzheimer’s disease have discovered that amyloid-β protein (Aβ) increases phospho-S880 on GluR2 subunits in hippocampal neurons, which can remove GluR2 from the cell surface [36]. Another study found GluR2/3 trafficking and activity of Src kinase in the IR condition [37]. In the 15 min of global ischemia model followed by 0.5 h, 4 h, and 24 h of reperfusion, Zhang et al. found that Src kinase was activated as a core glutamate receptor kinase after IR injury in GluR2/3 [37]. Additionally, GluR2/3 trafficking was related to the phosphorylation site S880, which was significantly up-regulated after IR injury. By contrast, inhibition of Src kinase protects the brain from ischemic injury. Such studies are few, but they do lend supporting evidence of changes to pGluR2 subunits at S880 during propofol post-conditioning, and the trafficking of AMPARs. In our study we showed that S880 was phosphorylated during IR injury, while propofol (20 mg/kg/h) reduced the phosphorylation level of GluR2, which might contribute to the stabilization of AMPARs on neuronal surfaces. Our results also demonstrated that the prevention of both trafficking and phosphorylation of GluR2 subunits caused by propofol can be reversed by LY294002, which suggested that PI3K may be the upstream signaling pathway of GluR2 subunits and responsible for the trafficking and phosphorylation of GluR2. The limitation of the current experiment is that we haven’t study the relationship and consequence between the phosphorylation of GluR2 and trafficking of this subunit, which means the change of GluR2 trafficking when the phosphorylation of GluR2 was blocked.

### PI3K and its regulating role of GluR2 subunits

In 1985, PI3 kinases were first identified by Lewis Cantley and his colleagues, and were shown to be capable of phosphorylating the 3′ hydroxyl group of the inositol ring of phosphatidylinositol (PtdIns) to produce the novel phosphoinositide PtdIns(3,4,5)P3 from PtdIns(4,5)P2 [38]. The PI3K/AKt pathway plays an important role in protecting the brain from a lot of disorders such as ischemia, hypoxia, and free radical induced oxidative stress. The PI3K/Akt pathway is well known for its involvement in synaptic plasticity, and memory consolidation although the relationship between PI3K and long-term potentiation (LTP) is still debated. Among them, protein synthesis and AMPAR insertion in late-phase LTP requires activation of the PI3K pathway. And the activation of the PI3K pathway also takes effect for the maintenance of LTP in hippocampal CA1 neurons [39]. Consistent with its important role in LTP, the PI3K–Akt pathway is also known to induce protein synthesis and AMPAR membrane translocation [40], and previous studies have found that the PI3K/Akt pathway plays a crucial role in the post-conditioning provided by propofol [3]. Other studies support the positive role of PI3K in the indirect trafficking of AMPARs [41], since inhibition of PI3K in neurons reduced the GluR2 surface expression. Moreover, depletion of PIKE (phosphoinositide 3-kinase enhancer), which directly interacts and enhances the activity of PI3K and Akt, reduced the activity of GluR2-associated PI3K, and thereby reduced GluR2 surface retention. Arendt et al. [11] concluded that the continuous low expression of PIP (3), which is the product of PI3K at the postsynaptic terminal, is necessary for the stabilization of AMPARs. Consequently, the activity of PI3K is vitally important to the stable expression of GluR2 at the postsynaptic terminal. In our current research, we discovered that enhanced complexes between PI3K and AMPARs GluR2 subunits played a critical role in the post-conditioning provided by propofol, and the trafficking of GluR2 to the plasma membrane of neurons during the protection, while the total expression of GluR2 subunits did not change a lot. The trafficking of GluR2 subunits was regulated by PI3K, for which the addiction of LY294002 would weaken the location of GluR2 subunits on the cytomembrane and postsynaptic membrane.

Our co-immunoprecipitation results showed that PI3K, which consists of both a catalytic (p110) and a regulatory (p85) subunits [42], enabled connection of the GluR2 subunits via the p85 subunits, which was concordant with the previous observation for LTP, wherein PI3K connected with GluR2 subunits by the C-terminal [12].

## Conclusions

Propofol post-conditioning at 20 mg/kg/h for 2 h significantly increases the frequency of GluR2 subunits in the postsynaptic membrane of neurons and reduces the levels of phosphorylation. Additionally, the protection and changes to the status of PI3K–GluR2 can be reversed partly by the inhibitor of PI3K, LY294002. In consideration of the trafficking and the change of pGluR2 subunits partly depend on the activity of PI3K, we inferred that the propofol post-conditioning protection may triggered by the activation of PI3K and achieved by the transport of GluR2 subunits to the postsynaptic membrane as well as the lower level of phosphorylation. This conclusion can better guide the clinical application of propofol. The limitation of the current experiment was that is did not study the relationship and consequence between the phosphorylation of GluR2 and the trafficking of this subunit. Furthermore, the role of AMPARs GluR2 subunit which accumulated on the membrane of intracellular organelles, such as lysosomes, mitochondria, and Golgi Complexes should be addressed in our future work.

## Data Availability

The datasets generated during the current study are available from the corresponding author on reasonable request.

## Abbreviations

AMPAR: amino-3-hydroxy-5-methylisoxazole-4-propionic acid receptor
PI3K: phosphoinositide-3-kinase
GluR: glutamate receptor
MCAO: middle cerebral artery occlusion
bFGF: fibroblast growth factor
Akt: protein kinase B
mNSS: modified neurological severity score
TTC: triphenyltetrazolium chloride
LTP: long-term potentiation
LTD: long-term depression
PtdIns-3P: phosphoinositide-3,4,5-trisphosphate
PDZ: protein 95/discsslarge/zon occlusens-1
S880: serine 880
PtdIns: phosphatidylinositol
PIKE: phosphoinositide 3-kinase enhancer
pGluR: phosphorylated glutamate receptor
IR: ischemia–reperfusion
